# Nephrotoxicity of High and Conventional Dosing Regimens of Colistin: A Randomized Clinical Trial

**Published:** 2017

**Authors:** Atefeh Ordooei Javan, Shervin Shokouhi, Zahra Sahraei, Jamshid Salamzadeh, Saeed Azad Armaki

**Affiliations:** a *Department of Clinical Pharmacy, School of Pharmacy, ShahidBeheshti University of Medical Sciences, Tehran, Iran.*; b *Department of Infectious Disease, Loghman Hospital, ShahidBeheshti University of Medical Sciences, Tehran,Iran.*; c *Department of Clinical Pharmacy, School of Pharmacy, ShahidBeheshti University of Medical Sciences, Tehran, Iran. *; d *Department of Clinical Pharmacy, School of Pharmacy, ShahidBeheshti University of Medical Sciences, Tehran, Iran.*; e *Pathologist director of Khatam laboratory, Khatam hospital, Tehran, Iran.*

**Keywords:** Colistin, Conventional dose, Cystatin C, High dose, Nephrotoxicity

## Abstract

Nephrotoxicity has been a major long-standing concern about colistin.This study was designed to compare nephrotoxicity of high dose and conventional dose of colistin.

A randomized open-labeled clinical trial on 40 patients with multi-drug resistant gram negative infections was designed. Patients were allocated into two equal-size groups receiving high (a loading dose of 9 million international units (MIU) and maintenance doses of 4.5 MIU every 12 h) and conventional dose (2 MIU every 8 h) of colistin. Blood samples were taken on day 1, 3, 5, 7 and 10 of treatment for measuring serum cystatin C (Cys C) levels. Incidence of acute kidney injury (AKI) was also evaluated based on RIFLE criteria.

Mean ± sd of the difference between baseline and day 10 Cys C levels in high dose and conventional dose groups were 1.61 ± 0.90 and 1.32 ± 0.48, respectively (P = 0.30). Within group analysis showed increase in Cys C levels in both groups (P = 0.001),however, no significant difference in Cys C levels was seen in between groups analysis (P = 0.13). Prevalence of AKI based on RIFLE criteria was 60% and 15% in high dose and conventional dose groups, respectively (P = 0.003). Comparison of Cys C between AKI (mean ± sd) and non-AKI (mean ± sd) patients, irrespective of colistin dosage regimens, confirmed a significant difference (P < 0.0001). Although, colistin-induced nephrotoxicity, determined by Cys C levels, was not confirmed by our findings, however, higher incidence of AKI in high-dose group, defined by RIFLE criteria, along with higher levels of Cys C in AKI patients are supportive of the higher risk of renal toxicity associated with high-dose regimen of colistin. More RCTs with a larger sample size are recommended.

## Introduction

Colistin, discovered in 1949, is a glycopeptideantibiotic which isproduced by a certain strain of *Bacillus polymixa* variant called colistinus(1). Although, colistin was available for clinical use in 1960s, however, due to concerns about its toxicity in particular nephrotoxicity, it was replaced by less toxic antibiotics almost a decade after its introduction to the market (2, 3). In the past 10-15 years, the emergence of multi-drug resistant (MDR) gram negative bacilli, especially *Pseudomonas aeruginosa*, *Acintobacterbaumannii* and *Klebsiella pneumonia*, along with the drying antibiotic development pipeline have led to a worldwide increase in reconsidering the use of colistin(2-4).

**Table 1:** RIFLE criteria

**RIFLE criteria**		
	**Serum creatinine (S Cr) criteria**	**Urine output (UO) criteria**
Risk	↑ S Cr × 1.5	< 0.5 mL/Kg/h × 6 h
Injury	↑ S Cr × 2	< 0.5 mL/Kg/h × 12 h
Failure	↑ S Cr × 3 Or ≥ 0.5 mg/dL if baseline S Cr ↑ to > 4 mg/dL	< 0.3 mL/Kg/h × 24 h or anuria × 12 h
Loss	Complete loss of renel function > 4 weeks	
End stage	End stage renal disease for > 3 month	

**Table 2:** Patients’ characteristics and clinical features of infectious episodes

** Groups **	**High dose group**	**Conventional dose group**	**P-value**
**Variable**			
Age (mean ± sd)	60.95(12.77)	57.8(21.85)	0.58
Male (%)	12(60)	15(75)	0.50
APACHE II score on the first day (mean ± sd)	18.5(5.88)	17.35(6.11)	0.55
Site of infection			0.29
Lung (%)	14(70)	14(70)	
Blood (%)	4(20)	6(30)	
CNS (%)	2(10)	0	
Pathogen type			0.61
Acinetobacterbaumannii(%)	18 (90)	17(85)	
Pseudomonas aeruginosa (%)	0	2(10)	
Klebsiella pneumonia (%)	2(10)	1(5)	
Baseline serum creatinine level (mean ± sd)	0.9(0.2)	0.89(0.17)	0.86
Baseline serum cystatin C level (mean ± sd)	1.1 (0.44)	0.9 (0.32)	0.11
Concomitant nephrotoxic drugs (mean ± sd)	0.8 (0.7)	0.65 (0.49)	0.60
Concomitant nephroprotective drugs (mean ± sd)	0.85 (0.49)	0.9 (0.85)	0.93

***APACHE II: Acute Physiology and Chronic Health Evaluation , sd: standard deviation***

**Table 3:** Comparison of RIFLE criteria between groups

	**No AKI**	**AKI**			**P-value**
		**RIFLE**			
** Risk category**	**None**	**Risk**	**Injury**	**Failure**	
**Study group**					
High dose group	8	3	7	2	0.01
Conventional dose group	17	2	1	0	

**Table 4.** Comparison of SrCr changes within and between groups

**Between group P value** 2	**Within group P value** 1	**SrCr (mg/dl)**					
0.02	0.00	**Day 10**	**Day 7**	**Day 5**	**Day 3**	**Baselie**	Dosing
		1.02 ± 0.37	0.96 ± 0.27	0.84 ± 0.26	0.90 ± 0.23	0.89 ± 0.17	Conventional dose
		1.77 ± 1	1.40 ± 0.77	1.20 ± 0.57	1.00 ± 0.32	0.90 ± 0.20	High dose
		0.01	0.01	0.01	0.21	0.86	P^3^

1 : Repeated measure Test.

2 : Repeated measurestest, comparing differences.

3 : Independent sample t- test

**Table 5:** Comparison of Cys C changes between high dose and conventional dose groups

**Between group P value** 2	**Within group P value** 1	**Cys C (mg/mL)**					
0.13	0.00	**Day 10**	**Day 7**	**Day 5**	**Day 3**	**Baseline**	Dosing
		1.32 ± 0.48	1.18 ± 0.44	1.03 ± 0.37	0.99 ± 0.38	0.90 ± 0.30	Conventional dose
		1.61 ± 0.90	1.63 ± 0.96	1.47 ± 0.98	1.20 ± 0.67	1.10 ± 0.44	High dose
		0.30	0.06	0.06	0.20	0.11	**P** 3

1 : Repeated measure test.

2 : Repeated measure test, comparing difference.

3 : Independent sample t- test

**Table 6:** Comparison of SrCr changes between AKI and non- AKI patients

**Between group P value** 2	**Within group P value** 1	**Creatinine (mg/dl)**					
0.00	0.00	**Day 10**	**Day 7**	**Day 5**	**Day 3**	**Baseline**	
		0.86 ± 0.27	0.88 ± 0.24	0.84 ± 0.26	0.86 ± 0.24	0.87 ± 0.19	**No AKI**
		2.18 ± 0.76	1.56 ± 0.72	1.31 ± 0.60	1.10 ± 0.28	0.93 ± 0.15	**AKI**
		0.00	0.00	0.00	0.00	0.30	**P** 3

1 : Repeated measure test.

2 : Repeated measure test, comparing difference.

3: Independent sample t- test

**Table 7:** Comparison of Cys C changes between AKI and non- AKI patients

**Between group P value** 2	**Within group P value** 1	**Cys C (mg/mL)**					
0.00	0.00	**Day 10**	**Day 7**	**Day 5**	**Day 3**	**Baseline**	
		1.08 ± 0.48	1.02 ± 0.41	0.97 ± 0.37	0.93 ± 0.36	0.89 ± 0.32	**No AKI**
		2.18 ± 0.60	1.59 ± 0.40	1.33 ± 0.47	1.15 ± 0.24	1.11 ± 0.28	**AKI**
		0.00	0.00	0.00	0.00	0.02	**P** 3

1 : Repeated measure test.

2 : Repeated measure test, comparing difference.

3 : Independent sample t- test

**Figure 1:**
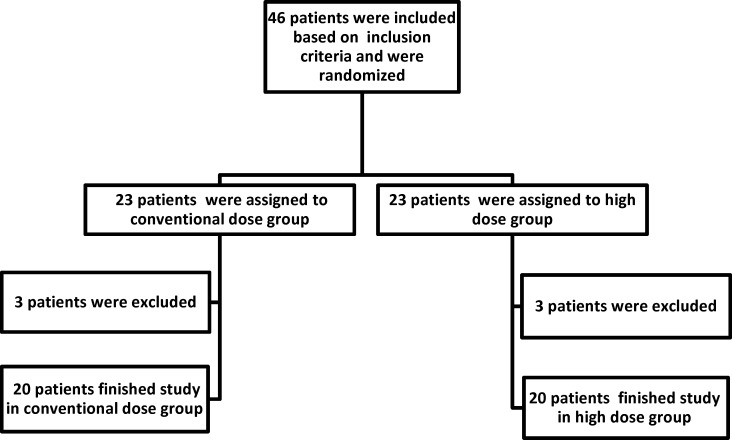
Study CONSORT

**Figure 2:**
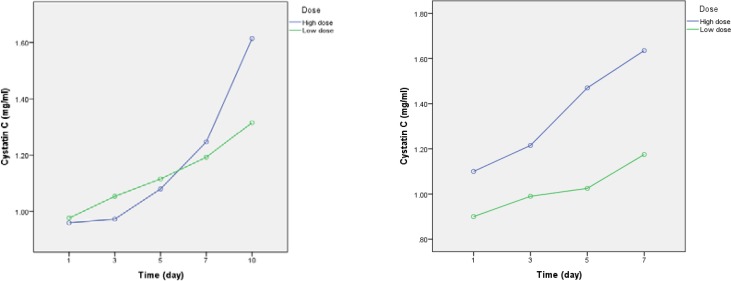
Serum Cystatin C level changes during studyperiod in high dose and conventional dose groups.a)Serum Cystatin C level changesuntill day 10. b) Serum Cystatin C level changesuntill day 7

**Figure 3:**
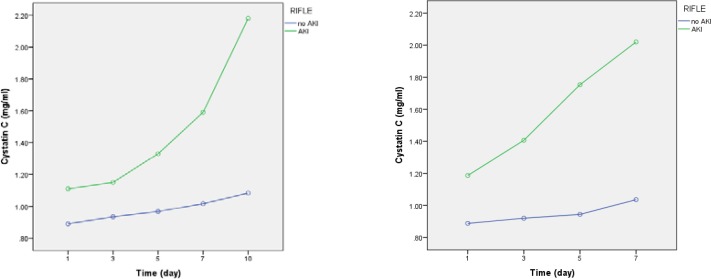
Serum Cystatin C level changes during study period in AKI and non- AKI groups.a)Serum Cystatin C level changesuntill day 10. b) Serum Cystatin C level changes untill day 7.

Along with reemergence of colistin, many complementary trials about different aspects of its utilization were carried out.Consequently, pharmacokinetic studies raised noteworthy questions about its conventional dosing. Problems with conventional doses are delay to reach steady state (suggestive of the necessitation of loading dose) and inadequate drug level to eradicate resistant pathogens (suggestive of higher maintenance doses). These ineffective low levels of drug not only may lead to failure of treatment but also enable the organisms to acquire resistance to the last-line of anti-infective drug therapy (5-7). The conclusion of pharmacokinetic model studieswas recommendation of a loading dose of 9 or 12 million units ofcolistimethate sodium (CMS) followed by maintenance doses of 4.5 million units twice daily (8-10). These high-dose regimens increased concerns about the nephrotoxicity of colistin leading to its limited administration. They also were suggestive of performing further studies to confirm efficacyandto define prevalence as well as severity of nephrotoxicity induced by colistin. Prevalence of colistin nephrotoxicity in a prospective study performed byDalfino and colleagues, the study that confirmsthe new dosing regimen, carried out on 28 patients receiving loading dose of 9 MIU and maintenance dose of 4.5 MIU twice-daily, was 17.8 % (11).

Adverse drug reaction profile of colistin generally include nephrotoxicity and neurotoxicity. Nephrotoxicity is more common and concerning adverse effect of colistin. Clinical manifestation of colistin nephrotoxicity is via a decrease in clearance of creatinine, proteinuria, cylindruria or oliguria (12-14). In a review article published in 2011, prevalence of colistin-induced nephrotoxicity was different ranging from 0 to 53.5%. Most of the trials included in this review were on low doses of colistin(15). 

Unfortunately, whathave beenconfusingaboutthe colistin areitsdosage formson the market. Commercially available forms of colistin are colistin sulfate for oral (intestinal decontamination) and topical use, and CMS for use via parenteral and inhalation routes. CMS is a less potent and less toxic prodrug of colistin(1, 16). It is converted to colistin *in-vivo *(2, 14). 

There are two most readily available brands of colistin for parenteral use as Coly-mycin® and Colomycin®, both of which contain CMS. However, the productsʹ labels are different and have led to mistakes. Colomycin® is labeled as 500,000, 1,000,000 and 2,000,000 IU of CMS, equivalent to 40, 80, and 160 mg of CMS, while, Coly-mycin® is labeled as 150 mg colistin base activity (CBA), equivalent to 5 Million International Unit (MIU) or 400 mg CMS(2). Therefore, due to the very different drug exposures in the two regimens, it is very important to know that which product is used in studies and that mentioned dose is based on CMS or CBA. 

Early detection of acute kidney injury (AKI) is a problem for early initiation of therapy. Currently serum creatinine (SrCr) is used for detection of AKI in clinical practice. Risk–Injury–Failure–Loss–End-stage renal disease (RIFLE) and Acute Kidney Injury Network (AKIN) classifications are usually used for evaluation of drug induced AKI in patients (Table1).Creatinine levels have some limitations forthe early and specific detection of AKI. These limitations include dependency of SrCr to age, sex, body mass, nutrition, and hydration status as well as delay in SrCr changes which is usually detected when about 50% loss in kidney function occurs. Another limitation is the fact that rises in creatinine secretion ratio at lower glomerular filtration rate may lead to overestimation of kidney function. Finally, during acute change in kidney function, in order to accurate estimation of glomerular filtration rate (GFR) based onSrCr, it is necessary to reach steady state which may need several days. Due to these limitations, several biomarkers for early detection of AKI have been introduced. 

Cystatin C (Cys C) is one of the novel biomarkers to predict kidney function. Cys C is a cysteine protease inhibitor synthesized at a constant rate by all nucleated cells. It is freely filtered by the glomerulus, catabolized completely in proximal tubules, but not secreted by the tubules. Unlike SrCr, the levels of Cys C are not affected by sex, age, race, or muscle mass. It seems to be useful as both serum or urinary biomarker (17, 18).

Considering the current arguments and uncertainty on nephrotoxicity risk induced by different dosage regimens of colistin, the presenthead to head clinical trial was designed.Wealso applied the Cys C as a biomarker for AKI detection and the RIFLE criteria to detect AKI.

## Experimental

*Materials and Methods*

The study was a prospective randomized open-labeled clinical trial to compare the nephrotoxicity of two conventional and high dosage regimens of colistin approved by the ethics committee of the ShahidBeheshti University of Medical Sciences and registered in the Iranian Registry of Clinical Trials (IRCT) with IRCT registration number of IRCT2014090614693N5. 

High dose regimen defined as a loading dose of 9 million international units (MIU) and maintenance doses of 4.5 MIU every 12 h (7, 10, 19) which was compared with conventional dosing of 2 MIU every 8 h. Brand and dosage form of colistinused in this study was “Colomycin®” vials containing 1 MIU of sodium colistimethate (CMS).

*Study population*

Patients, age > 18 years old, with a multi-drug resistant gram negative infection (candidate to receive colistin therapy) admitted into a teaching hospital affiliated to the ShahidBeheshti University of Medical Sciences, Tehran, Iran, entered the study. Exclusion criteria were: patients with chronic kidney disease (GFR < 50), acute kidney injury, BMI > 35 and pregnancy. 40 patients were randomly allocated into two equal-size groups during May 2014 to January 2015.

*Sampling time and measurements*

Blood samples were obtained on days 1 (as baseline before first dose), 3, 5, 7 and 10 of treatment. Separated serums were stored at -70 °C. Daily SrCr measurements were performed until day 10 and patients were observed for occurrence of AKI based on RIFLE (Table 1) criteria through the study period.

*Patients’ Data*

Patients’ characteristics and medical status including age, sex, Acute Physiology, and Chronic Health Evaluation (APACHE) II score on the first day of colistin therapy, type of infection, causative organism of infection and in vitro susceptibility were recorded. Co-administered antibiotics, nephrotoxic agents as well as drugs with renal adverse effects (aminoglycosides, vancomycin, non-steroidal anti-inflammatory drugs, intravenous radio contrast agent, diuretics, mannitol) were also documented.

*Laboratory analytical methods*

Cys C concentration measurement was performed by TURBILATEX Cystatin C kit Biorex Diagnostics® (BXC0777- CYSTATIN-C, United Kingdom). Using this kit, Cys C assay is based on a latex enhanced immunoturbidimetry assay. Cys C in the sample binds to the specific anti-Cystatin C antibody, which is coated on the latex particles and causes agglutination. The degree of turbidity is directly proportional to the concentration of Cys C in the sample and is measured photometrically. Calculation of concentration is performed by interpolation of the absorbance on a 6 point calibration curve.

*Statistical analysis*

Statistical analysis was performed using the SPSS software (Statistical Package for the Social Sciences, version 21, SPSS Inc, Chicago, Ill, USA). Continuous normally distributed data are expressed as mean (± SD) and compared using the unpaired Student t-test. Non-normally distributed data are expressed as median and interquartile range (IQR) and compared using the Mann-Whitney U test. Categorical data are expressed as number and percentage of events and compared using the Fisher’s exact test. For repeated measurements of SrCr and Cys C, a General Linear Model Repeated Measures analysis was applied. In all comparisons, a P value < 0.05 was considered statistically significant.

## Results

*Demographic data*

Out of 46 patients who were enrolled in this study, 6 patients could not complete the study and were excluded in the 1st week of colistin therapy (3 patients in high dose group and 3 patients in conventional dose group) and 40 patients fulfilled the inclusion criteria and completed the study. Study flowchart was shown in Figure 1.

Patients were predominantly males (12(60%) in high dose group and 15(75%) in conventional dose group) (p = 0.50).Mean±sd age was 60.95 ± 12.77 years in high dose group and 57.8 ± 21.85 years in conventional dose group (p = 0.58). The major infection type was Ventilator Associated Pneumonia (VAP) or Hospital Acquired Pneumonia (HAP), with a frequency of 70% in each group. Mean APACHE II score was 18.5 ± 5.88 in high dose group and 17.35 ± 6.11 in conventional dose group (p = 0.55). Baseline SrCr and Cys C levels were not significantly different between groups (p = 0.86 and p = 0.11, respectively). The patients’ characteristics and clinical features of their pulmonary infection are summarized in Table 2. There is not statistical difference between groups as shown in the Table 2, confirming appropriate randomization of the patients in the study groups.

*Comparison of RIFLE criteria between high dose and conventional dose groups*

During the 10 days of study period, prevalence of AKI based on RIFLE criteria was 60% (12 out of 20 patients) and 15% (3 out of 20 patients) in high dose and conventional dose groups, respectively. The difference was statistically significant (p = 0.01) (Table 3), so thatincidence and severity of AKI was significantly greater in high dose colistin therapy compared to conventional dose therapy (Table 3). 

*Comparison of serum Creatinine levels between high dose and conventional dose groups*

Comparison of the SrCr differences between groups was done with Repeated Measures analysis. SrCr level increased notably in both groups from first day of study (before Colistin therapy) to end of the study (p = 0.002). This elevation was also significantly higher in patients who have received high doses of colistin (p = 0.02). Paired comparison of the SrCr levels in different sampling days of 3, 5, 7 and 10 revealed that from day 5, there were significant differences between two groups (Table 4).

*Comparison of serum Cystatin C levels between high dose and conventional dose groups*

Baseline levels of Cys C were not statistically different between patients in two arms of the study. Although, within group analysis confirmed a significant increase in Cys C levels from day 1 to day 10 of the study (p = 0.001), however, between groups analysis did not show a significant difference in the Cys C levels. (p = 0.13) (Table 5 and Figure 2).As a complementary analysis, we compared the occurrence and severity of AKI based on RIFLE criteria, irrespective of dosage regimen, in the study patients (n = 40). In these analyses, dosing regimen itself was considered as a covariate. Overall, 15 (37.5%) out of 40 patients developed AKI. The relevant analyses are presented below.

*Comparison of serum Creatinine levels between AKI and non- AKI patients*

Table 6 presents the results of this analysis. As it is seen, from day 3 (p = 0.004) to day 10 (P < 0.001), SrCr levels are significantly higher in the AKI patients compared to non-AKI patients.

*Comparison of serum Cystatin C levels between AKI and non- AKI groups*

As illustrated in Table 6, from day 1 (p = 0.002) to day 10 (p < 0.001), Cys C levels are significantly higher in the AKI patients compared to non-AKI patients (Table 7, Figure 3).

## Discussion

This study was an approach to compare the nephrotoxicity effect of high dose colistin regimen with that of conventional regimen. Cys C was used as a biomarker for early detection of kidney injury caused by colistin.At the same time, RIFLE classification of AKI was applied to describe the changes in SrCr and Cys C levels in AKI and non-AKI patients. Currently, nosocomial multi drug resistant gram negative infections are one of the major concerning problems in hospitals.This lead to the re-emerging of colistin, an antibiotic which withdrew from the market because of its nephrotoxicity several years ago (20). 

Pharmacokinetics studies on colistin suggested that one need to use higher loading and daily doses of the drug to accelerate achievement of the target levels.Dose dependent colistin nephrotoxicity still is a major concern that has forced limitation in its usage. In this regard, studies to evaluate prevalence of colistin nephrotoxicity, and its early detection and prevention arerecommended. Our study was designed to assess the incidence of nephrotoxicity with high and conventional doses of colistin regimens in a head to head clinical trial.

Prevalence of AKI based on RIFLE criteria was 60% (12 out of 20 patients) and 15% (3 out of 20 patients) in high dose group and conventional dose group, respectively. Occurrence of AKI was significantly higher in high dose regimen group and the difference in serum creatinine between groups was significant from day 5 of colistin therapy. Mean start day of AKI was 6.5 ± 2.6 days and 7.3 ± 0.58 days for high dose and conventional dose groups, respectively. 9 (60%) patients out of the 15 patients, developed AKI, within 7 days after colistin therapy and the remaining 6 patients (40 %) developed AKI after 7 days. 

In a study, KO *et al*. divided AKI into early (within 7 days) and late (after 7 days) AKI groups. They showed patients with early AKI had higher in-hospital mortality than those with late AKI. Prevalence of AKI with colistintherapy was 54.6%, of which approximately 70% developed early AKI. They concluded that early occurrence of AKI might be fatal and careful monitoring of renal function is necessary. They also suggested further study to determine early markers of predicting AKI (21). 

In a retrospective study, Deryke *et al*. have shown that the prevalence of nephrotoxicity of colistin therapy (5.1 ± 2.4 mg/Kg/day) was 33% which was developed during the first five days of treatment (22). Pogue *et al*. has also reportednephrotoxicity rate of 43% amongst study patients (n = 126). 78% of themdeveloped AKI within 7 days of colistin therapy (23).

In a retrospective cohort study on 132 patients, Tuon *et al*. revealed that prevalence of AKI was 25.8 %. The median time to development AKI was 7.5 days (in the rage of 2 to 21 days)(24). 

Based on our results, start day of AKI was 6.5 ± 2.6 days and 7.3 ± 0.58 days for high dose and conventional dose groups, respectively. These findingsare verycomparable to those of other studies which were mentioned above.These emphasize the importance of early detection of colistin nephrotoxicity and its prompt management.

In our study, althoughincrease in Cys C levelswas higher in the high dose group compared to conventional dose group, however it was notstatistically significant. In contrast, within group analyses approved a significant increase in Cys C levels in both groups from day 7.

These findings confirm that the infectious disease itself or any unknown variations existing between patients included in each arm of the study have acted as confounding factors influencing change in Cys C levels. In other word, controversy on recognition of Cys C as an accurate and sensitive biomarker for early detection of AKI is still remaining.Comparing AKI and non-AKI patients, irrespective of the difference in dosage regimens, showed that Cys C levels were significantly higher in the AKI group from the 1^st^ day of colistin use, while the levels of SrCrin AKI patients were higher than that of non-AKI patients from the 3rd day of colistin therapy. This is likely due to the fact that rise in serum Cys C is 48 h before rise in SrCr in AKI patients, as reported by previous studies (25, 26).

## Conclusion

Although, colistin-induced nephrotoxicity, determined by Cyc C levels, was not confirmed by our findings, , higher incidence of AKI in high-dose group, defined by RIFLE criteria, along with higher levels of Cys C in AKI patients are supportive of the higher risk of renal toxicity associated with high-dose regimen of colistin. Further studies to explore novel accurate and sensitive biomarkers for determining the early deterioration of renal function in patients receiving colistinare recommended.
